# GPAT: Retrieval of genomic annotation from large genomic position datasets

**DOI:** 10.1186/1471-2105-9-533

**Published:** 2008-12-15

**Authors:** Arnaud Krebs, Mattia Frontini, Làszlò Tora

**Affiliations:** 1 Department of Functional Genomics, Institut de Génétique et de Biologie Moléculaire et Cellulaire (IGBMC), CNRS UMR 7104, INSERM U 596, Université Louis Pasteur de Strasbourg, BP 10142-67404 ILLKIRCH Cedex, CU de Strasbourg, France

## Abstract

**Background:**

Recent genome wide transcription factor binding site or chromatin modification mapping analysis techniques, such as chromatin immunoprecipitation (ChIP) linked to DNA microarray analysis (ChIP on chip) or ChIP coupled to high throughput sequencing (ChIP-seq), generate tremendous amounts of genomic location data in the form of one-dimensional series of signals. After pre-analysis of these data (signal pre-clearing, relevant binding site detection), biologists need to search for the biological relevance of the detected genomic positions representing transcription regulation or chromatin modification events.

**Results:**

To address this problem, we have developed a **G**enomic **P**osition **A**nnotation **T**ool (GPAT) with a simple web interface that allows the rapid and systematic labelling of thousands of genomic positions with several types of annotations. GPAT automatically extracts gene annotation information around the submitted positions from different public databases (Refseq or ENSEMBL). In addition, GPAT provides access to the expression status of the corresponding genes from either existing transcriptomic databases or from user generated expression data sets. Furthermore, GPAT allows the localisation of the genomic coordinates relative to the chromosome bands and the well characterised ENCODE regions. We successfully used GPAT to analyse ChIP on chip data and to identify genes functionally regulated by the TATA binding protein (TBP).

**Conclusion:**

GPAT provides a quick, convenient and flexible way to annotate large sets of genomic positions obtained after pre-analysis of ChIP-chip, ChIP-seq or other high throughput sequencing-based techniques. Through the different annotation data displayed, GPAT facilitates the interpretation of genome wide datasets for molecular biologists.

## Background

One of the major issues in genomics is the genome wide mapping of transcription factor binding sites in order to study their function at the scale of the genome. The chromatin immunoprecipitation (ChIP) technique uses antibodies that are specific for a transcription factor or a chromatin modification, to isolate the DNA to which this factor or modified histone is bound in a cell at a given time. The recent appearance of several genome wide analysis techniques, where ChIP is either followed by DNA microarray analysis (ChIP on chip) or coupled to high throughput sequencing (ChIP-seq), made the genome wide mapping of DNA bound factors technically possible. However, these analyses generate tremendous amounts of genomic location data in the form of one-dimensional series of signals.

Recently, efforts have been made to develop academic software to pre-analyse these datasets, (e.g. Mpeak [1]), in order to locate the signal peaks that correspond to functional elements, such as promoters, enhancers, repressors or insulators. However, once these datasets are cleaned and the significant signals are selected, biologists lack user-friendly tools to search for the biological relevance of the resulting binding site genomic positions.

## Implementation

The web interface of GPAT is programmed using Python (v2.5.1) [2] running on an Apache [3] WWW server and forms an interactive layer between the user and the underlying processing applications. In order to increase the speed of data recovery, all the data are stored in a local PostgreSQL (v8.1.11) database [4]. The background processes are programmed in Python and take advantage of the PygreSQL [5] module to efficiently connect to the PostgreSQL database. The class diagram describing the GPAT object oriented python code is displayed in Additional file 1: GPAT class diagram.

## Results

### Comparison with existing tools

Existing on-line tools, such as the UCSC genome browser [6], allow the user to display so-called "custom tracks" and to browse locally defined genomic positions, thus facilitating the manual retrieval of biological information over hundreds of annotation tracks. However, the browser does not allow the batch processing of large numbers of positions. Complementary to this approach, studies based on genome wide analyses require a tool to systematically annotate data in a form suitable for further statistical analysis. To address this problem, several applications have been released recently.

Galaxy [7] is a framework giving access to popular sources of data, such as the UCSC Table Browser [6] or Biomart at ENSEMBL [8], using a variety of integrated tools. Although very powerful for certain applications, it remains too general for our purposes. In particular, the "Fetch the closest feature" module in the "Operate on genomic intervals" section that allows the annotation of genomic positions with gene identifiers has some limitations compared to a more specialized tool, such as GPAT (see below). As an example, the "Fetch the closest feature" module searches for the closest feature without distance limitations, and often matches very distant genes with questionable biological relevance. In addition, the output is limited to a plain text file corresponding to the concatenation of the input files with restricted information content (e.g. the distance to the TSS is not calculated and no hyperlinks to gene annotations are provided). Moreover, the direct cross-linking of the identified gene list with transcriptomic datasets is not directly possible.

CEAS [9] is a ChIP on chip analysis pipeline available via a web server, which includes a basic genomic position annotation function. One of the major limitations of CEAS is that the annotation search is completely automated and the user cannot adjust the default options. For example, the search window around the submitted position is fixed at 300 bp. This means that the software is not suitable for studies involving factors, which are known to bind enhancer regions located outside of the proximal promoter regions. Furthermore, since the full analysis is done at each dataset submission, the analysis time rapidly increases for large datasets (20 minutes for 1000 positions).

Cisgenome [10] is a powerful high throughput ChIP analysis package proposing a gene annotation function. However, the user has to perform a full installation process and must store the annotation files on a local computer (several gigabytes) in order to annotate his results. Furthermore, Cisgenome does not provide direct access to other annotations and experimental data (e.g. transcriptomic data), which is a necessary step in the extraction of biological meaning from high throughput data.

### GPAT description

**Figure 1:**
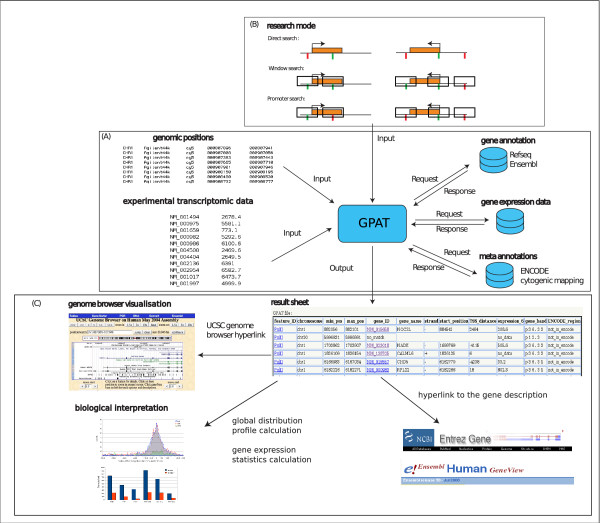
**GPAT application flow chart**: (A) Information flow of an annotation search in GPAT. (B) The three gene annotation search modes implemented in GPAT. The panel represents two transcription units oriented in opposite directions (orange boxes). The transcription start site (TSS) is symbolised by an arrow. User submitted positions are represented by vertical bars and the search window by open boxes. The colour of the vertical bar symbolizes the result of the GPAT search (green: annotation matched, red: not matched). The "direct search" mode searches the positions located inside a transcription unit. The "window search" mode allows the detection of transcription units located within a defined distance from the genomic positions. The "promoter search" mode allows the identification of transcription units having their TSS within a defined distance from the genomic positions. (C) Results table containing the annotated positions; links to UCSC genome browser and gene source information; global distribution profile of the matched genomic positions as compared to the TSSs of the corresponding genes and statistical values for the expression data of the corresponding genes (represented using a spreadsheet application).

GPAT allows users to analyse large batches of genomic positions and to retrieve genomic annotations around these positions. Briefly, the user submits a flat file containing the genomic positions and then selects the annotation search options and the display options (Figure 1A). The annotated results can then be browsed or downloaded for subsequent analyses.

The input format is based on the GFF standard file format [11]. Mouse and human data produced by the two latest UCSC genome assembly releases (mm 8, mm 9, hg 17, hg 18) are currently supported.

For each submitted genomic position, GPAT calculates a mean value from the two boundary positions and compares this value to the gene annotation positions (Refseq release 31, ENSEMBL release 50). GPAT has three different gene annotation search modes corresponding to different biological questions (Figure 1B).

1. The "direct search" mode determines whether the submitted positions are detected inside a transcription unit. It was developed to allow searches for a binding site inside the transcription units (e.g. retrovirus insertion events).

2. In the "window search" mode, a user-defined window is calculated around the submitted positions. Then GPAT searches gene annotations located within this window. It was developed for datasets where no particular binding profile is expected or known (e.g. insulator elements or proteins of unknown function).

3. The "promoter search" mode uses the same window as the "window search" mode, but tests whether the transcription start site (TSS) of a transcription unit is found within this window. It was developed for datasets, where a binding in the neighbourhood of the TSS is expected (e.g. transcription factors).

Regardless of the search mode chosen by the user, GPAT provides a complete localisation report relative to the matched gene annotation (position of the TSS of the corresponding gene, distance of the detected location to the TSS). Furthermore, when multiple gene matches are found, the user can choose to retrieve either the closest annotation only or all the matched annotations.

Biologists are often interested in the expression status of the genes neighbouring the submitted genomic locations. Therefore, the GPAT software also gives access to the corresponding gene expression levels in several commonly used model cell lines as provided by the GNF Symatlas [12] and Stembase [13] (nine human and two mouse cell lines including mouse embryonic stem cells). Furthermore, users can upload their own transcriptomic data during the analysis process, thus allowing the retrieval of gene expression levels for datasets generated under various experimental conditions.

Several other genomic features, such as those extracted from the cytogenetic mapping [14] or the position relative to the ENCODE regions [15] can also be retrieved.

GPAT displays the results as a table containing the annotated positions hyperlinked to the data source (Figure 1C). A direct hyperlink to the UCSC genome browser [6] is provided to allow the user to browse other genomic features in the case of a successful match. In addition, the full set of results can be downloaded as a tabulated flat file and easily imported in any spreadsheet software for further analysis.

Finally, an additional analysis step allows the user to create a report file, containing summary information, in addition to the matched gene list. This option calculates the global distribution profile of the matched genomic positions as compared to the TSSs of the corresponding genes using parameters set by the user (see example in Figure 1C). Statistical values for the expression data of the corresponding genes are also calculated (Figure 1C).

### Use of GPAT – Example:

The Tata Binding Protein (TBP) is a component of a number of complexes, including the TFIID complex involved in the RNA Polymerase II (Pol II) general transcription machinery. Surprisingly, it has been shown that Pol II transcription can occur in the absence of TBP [16,17]. However, the molecular mechanism leading to TBP-free transcription is still poorly understood. One of the strategies for studying this mechanism is to isolate target genes where Pol II transcription can be detected in the absence of TBP.

We tested GPAT using a promoter DNA tiling array dataset generated by hybridization of DNA (prepared from Hela cells), which was ChIPed using specific antibodies against Pol II, TBP and GST (as a negative control). For each experiment, three slides (Agilent G4483A – 013863, 013864 and 01387) representing approximately one-third of the most characterised promoters of the gene were hybridized (about 5600 genes covered from -8 kb upstream to +2.5 kb downstream with a ~350 bp resolution). After intra-array lowess normalization, peaks corresponding to the factor binding sites were detected using Agilent ChIP analytics (using the neighbourhood model).

**Figure 2:**
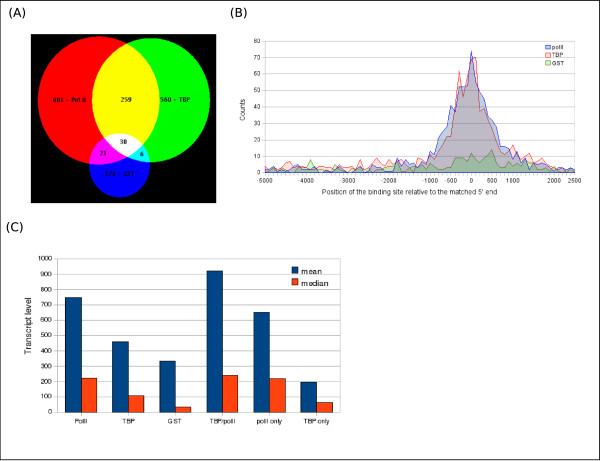
**Example of exploitation of the GPAT results**: (A) Venn diagram showing the genes with a single occupancy by Pol II (red) or TBP (green) respectively or a co-occupancy (yellow). (B) Distribution of Pol II (blue) and TBP (red) binding sites relative to the 5' end of the matched transcript. The distribution patterns of both Pol II and TBP, but not GST, cluster within +/- 1 kb around the 5' end of the matched transcripts. (C) Distribution of the expression level in each gene category. The highest expression level is observed for genes where both Pol II and TBP were detected at the promoter. Furthermore, genes bound only by Pol II, but not TBP show a high level of expression, suggesting the possibility of TBP independent genes.

A flat file containing the genomic positions generated by this pre-analysis was input to GPAT. The promoter search mode using a half window size of 5000 bp successfully retrieved the genes in the neighbourhood of the factor binding sites in less than a minute. This allowed us to build a list of genes potentially regulated by TBP and to obtain information about the presence of the Pol II at these sites (Figure 2A). Furthermore, using the distance to the 5' end of the matched gene transcripts provided in the GPAT output, the global distribution of the binding sites of TBP and Pol II relative to the 5' end could be computed (figure 2B). This shows, as expected, that the majority of the TBP and Pol II (but not GST) binding sites are located within +/- 1 kb of the TSS. This result is in agreement with observations made at the single gene scale and demonstrates the accuracy of GPAT results.

Finally, the output of GPAT allowed us to attribute an expression level for each of the differently regulated gene categories showing that the genes for which a co-occupancy of TBP and Pol II was detected have the strongest expression levels (Figure 2C). This correlation gives a high level of confidence in the list of binding sites established by our analysis. Furthermore, by combining these data, we obtained a list of expressed genes, for which Pol II, but not TBP is present (Figure 2A). These genes can be used as a model for further analysis of the mechanism of TBP-free transcription initiation. Finally, the group of genes for which TBP, but not Pol II is bound at the promoter shows low expression levels, implying that there are genes where TBP is bound, but Pol II, is not or is poorly recruited.

In conclusion, these results demonstrate the efficiency of GPAT for the extraction of biological meaning from large genomic position datasets.

## Discussion

The current version of GPAT improves the bioinformatics analysis of ChIP on chip or ChIP-seq data. It considerably speeds up and facilitates the steps between data pre-analysis and the biological interpretation of the data. Notably, the amplitude in the stringency of the annotation search provided by the three different research modes should meet the requirements of most of the biological questions addressed. Furthermore, the full localisation report (including distance to the TSS) will help the biologist to easily understand the binding pattern of his studied factors. Finally, the connection of the binding sites and the gene expression data is an essential resource at different steps of the analysis. Firstly, it provides a supplementary filtering step to distinguish between relevant and background signals. Secondly, it adds another layer of complexity to the data interpretation, providing insight into the regulation processes taking place at particular genomic locations.

Currently, the investigation process of large scale ChIP data is complicated and involves multiple analysis steps. In order to make this technology available for laboratories without access to bioinformatics expertise, considerable efforts are needed to facilitate the data analysis (similar to the efforts dedicated to classical expression arrays). To address this issue, GPAT was designed to improve the steps following data pre-analysis (annotation, cross-linking with other experimental data). However, several improvements remain to be implemented. GPAT currently supports the mouse and human genomes, but adding other model organisms for which genome wide ChIP data are available (e.g. Drosophila) would make the impact of GPAT wider and could be easily implemented in the future.

Furthermore, GPAT provides expression data for several cell lines. One of the recent improvements of the ChIP method allows the use of tissues as a starting material. Since the GNF dataset was also generated for mouse tissues, these datasets could be easily integrated into the GPAT interface.

## Conclusion

GPAT provides a quick, convenient and flexible way to annotate large sets of genomic positions obtained after pre-analysis of ChIP on chip, ChIP-seq or other high throughput sequencing based techniques. Thanks to the different annotation and experimental data provided (including the expression status of the identified genes), GPAT facilitates the interpretation of genome wide datasets. We hope that GPAT will be of great help to molecular biologists who wish to analyse large-scale genomic data.

## Availability and requirements

**Project home page **

Source code

The GPAT source code can be freely downloaded from: 

Operating systems

For use: Standard WWW browser

Programming language

Python, SQL, Javascript

Licence

GNU General Public License v3 (GPL 3)

## Abbreviations

ChIP chip: Chromatin Immunoprecipitation followed by hybridation on DNA microarray; ChIP-seq: Chromatin Immunoprecipitation coupled to high throughput Sequencing; ChIP: Chromatin Immunoprecipitation; GPAT: Genomic Position Annotation Tool; RNA Pol II: RNA polymerase II; TBP: TATA Binding Protein; TSS: Transcription Start Site

## Authors' contributions

AK conceived and implemented GPAT. MF generated the ChIP on chip data. AK did the bioinformatics analysis of the ChIP on chip data. AK and LT wrote the manuscript.

## Supplementary Material

Additional file 1 **GPAT class diagram**. Class diagram describing the python object oriented architecture of the GPAT software. Click here for file
