# Supplementary figures and images for: GPAT: Retrieval of genomic annotation from large genomic position datasets
Source: BMC Bioinformatics. 2008 Dec 15;9:533. doi: 10.1186/1471-2105-9-533 (PMC2654044; doi:10.1186/1471-2105-9-533)

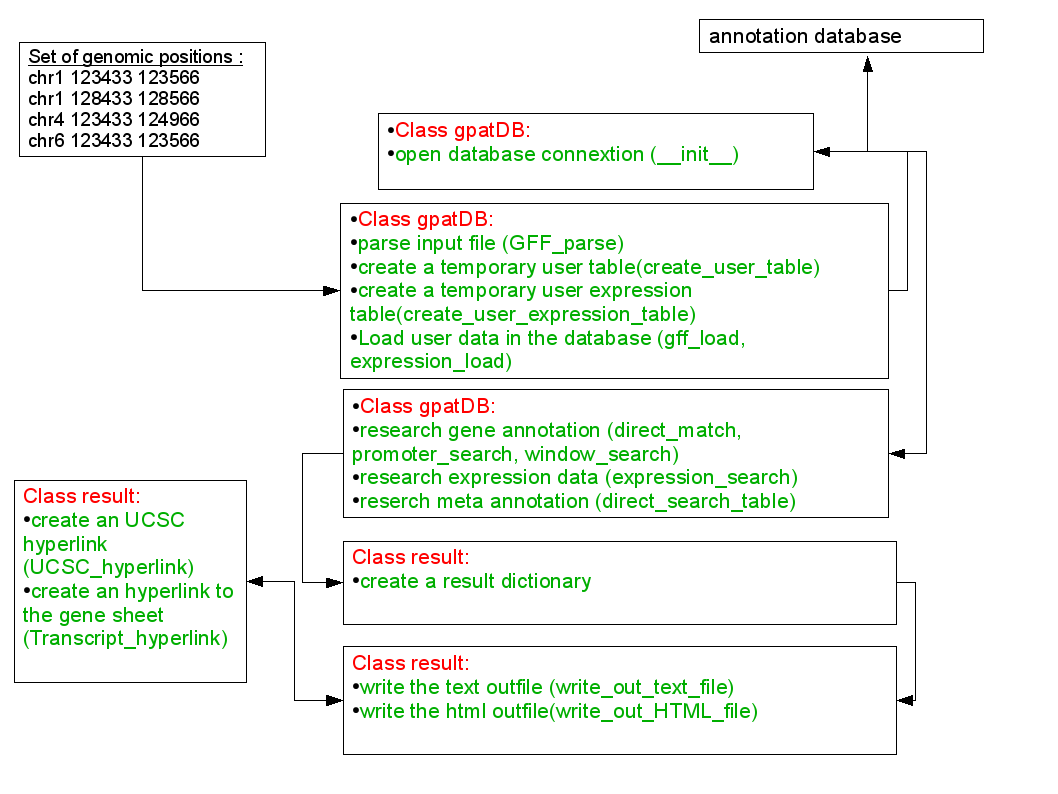

Supplement: Additional file 1 — GPAT class diagram. Class diagram describing the python object oriented architecture of the GPAT software. [file 1471-2105-9-533-S1.png]
